# Mobile App–Reported Use of Traditional Medicine for Maintenance of Health in India During the COVID-19 Pandemic: Cross-sectional Questionnaire Study

**DOI:** 10.2196/25703

**Published:** 2021-05-07

**Authors:** N Srikanth, Rakesh Rana, Richa Singhal, Sophia Jameela, Rajeshwari Singh, Shruti Khanduri, Arunabh Tripathi, Sumeet Goel, Leena Chhatre, Ashwin Chandra, B C S Rao, K S Dhiman

**Affiliations:** 1 Central Council for Research in Ayurvedic Sciences Ministry of AYUSH Delhi India; 2 Ministry of AYUSH Government of India Delhi India

**Keywords:** AYUSH Sanjivani app, COVID-19, traditional medicine, Ayurveda, Siddha, Unani, homeopathy

## Abstract

**Background:**

India follows a pluralistic system for strategic and focused health care delivery in which traditional systems of medicine such as Ayurveda, yoga and naturopathy, Unani, Siddha, Sowa Rigpa, and homoeopathy (AYUSH) coexist with contemporary medicine, and this system functions under the Ministry of AYUSH (MoA). The MoA developed a mobile app, called AYUSH Sanjivani, to document the trends of the use of AYUSH-based traditional and holistic measures by the public across India. Analysis of the data generated through this app can help monitor the extent of the use of AYUSH measures for maintenance of health during the COVID-19 pandemic and aid effective health promotion and communication efforts focused on targeted health care delivery during the pandemic.

**Objective:**

The purpose of the study was to determine the extent of use of AYUSH measures by the public in India for maintenance of health during the COVID-19 pandemic as reported through the AYUSH Sanjivani mobile app.

**Methods:**

Cross-sectional analysis of the data generated through the Ayush Sanjivani app from May 4 to July 31, 2020, was performed to study the pattern and extent of the use of AYUSH-based measures by the Indian population. The responses of the respondents in terms of demographic profile, use pattern, and benefits obtained; the association between the use of AYUSH-based measures and symptomatic status; and the association between the duration of use of AYUSH-based measures and the outcome of COVID-19 testing were evaluated based on bivariate and multivariate logistic regression analysis.

**Results:**

Data from 723,459 respondents were used for the analysis, among whom 616,295 (85.2%) reported that they had been using AYUSH measures for maintenance of health during the COVID-19 pandemic. Among these 616,295 users, 553,801 (89.8%) either strongly or moderately agreed to have benefitted from AYUSH measures. Ayurveda and homeopathic measures and interventions were the most preferred by the respondents across India. Among the 359,785 AYUSH users who described their overall improvement in general health, 144,927 (40.3%) rated it as good, 30,848 (8.6%) as moderate, and 133,046 (40.3%) as slight. Respondents who had been using AYUSH measures for less than 30 days were more likely to be COVID-19–positive among those who were tested (odds ratio 1.52, 95% CI 1.44-1.60). The odds of nonusers of AYUSH measures being symptomatic if they tested positive were greater than those of AYUSH users (odds ratio 4.01, 95% CI 3.61-4.59).

**Conclusions:**

The findings of this cross-sectional analysis assert that a large proportion of the representative population practiced AYUSH measures across different geographic locations of the country during the COVID-19 pandemic and benefitted considerably in terms of general well-being, with a possible impact on their quality of life and specific domains of health.

## Introduction

Coronaviruses, a large family of single-stranded RNA viruses, can infect animals and humans, causing respiratory, gastrointestinal, hepatic, and neurologic diseases [1]. To date, 6 human coronaviruses (HCoVs) have been identified, including the alpha coronaviruses HCoVs-NL63 and HCoVs-229E and the beta coronaviruses HCoVs-OC43, HCoVs-HKU1, and severe acute respiratory syndrome coronavirus (SARS-CoV) [2]. New coronaviruses appear to emerge periodically in humans owing to the high prevalence and wide distribution of coronaviruses, the large genetic diversity and frequent recombination of their genomes, and the increase of human-animal interface activities [3]. The first case of COVID-19 in India was reported on January 31, 2020 [4]. The World Health Organization observed that with appropriate integration, traditional medicine would be a significant option to balance curative services with preventive care, which can help address the unique health challenges of the 21st century [5].

Clinical evidence from a study on the effects of Chinese traditional medicine in the treatment of SARS-CoV-2 demonstrated significant results, and the study proposed that herbal medicine has a beneficial effect in the treatment and prevention of epidemic diseases [6]. A Cochrane systematic review in this area reported that herbal medicine combined with western medicine may improve symptoms and quality of life in SARS-CoV patients [7]. The National Health Commission in China has declared the use of herbal medicine combined with contemporary medicine as a treatment for COVID-19 and has issued many guidelines on herbal medicine–related therapy [8]. The acronym AYUSH stands for Ayurveda, yoga and naturopathy, Unani, Siddha, and homeopathy; these indigenous systems of medicine are practiced in India under the Ministry of AYUSH (MoA). Considering the present scenario and penetration of the AYUSH system into the mainstream health care system in India for preventive and curative purposes, the MoA released an advisory to the public for maintenance of general health and well-being during the COVID-19 pandemic on March 6, 2020 [9]. Although India is a country that follows a pluralistic approach to health care, data regarding the use of traditional systems of medicine or health-seeking trends of people are not available in the public domain. There are reports in the press regarding the use of AYUSH prophylactic measures for COVID-19 [10] as well as for lifestyle and other diseases; however, the extent of their use and the outcomes and benefits obtained are not known. Health care delivery, as well as research in times of natural disasters and epidemics or pandemics, is challenging [11]. The concept of infodemiology has evolved significantly with the ever-increasing penetration of the internet in society and is being efficiently being used to nowcast epidemics, quantify the different trends in epidemics, and document and synthesize data on the use of health care services and other public health–related issues [12,13].

The government of India has taken the initiative to use and integrate the preventive, curative, and rehabilitative potential of AYUSH systems of medicine to strengthen the health care delivery system, and the AYUSH Sanjivani app was developed through a consultative process among experts in the field of AYUSH and information technology (IT) by the MoA to record the patterns and trends of the use of preventive measures adopted by the public to enhance immunity and maintain health during the COVID-19 pandemic. The AYUSH Sanjivani app was intended to motivate and persuade users to achieve a status of healthy well-being while thwarting the tendency of the masses to use untested and unproven remedies or over-the-counter or self-prescription measures, especially when faced with the threat of the pandemic and the physical, physiological, social and economic ramifications of the containment measures required of the public.

Through recent initiatives in smart devices, mobile apps have become a convenient, easy-to-use, and less time-consuming method to generate data from the public. Self-reported health status and health care service use are indispensable indicators to assess the performance and attitude of any health system in the absence of recorded health administration data [14]. An app-based survey has advantages such as wider population access, better response rates, lower cost, ease of analysis, ease of use for participants, assurance of user anonymity and preferences, greater flexibility, and faster data synthesis compared to traditional epidemiological and surveillance methods. Various previous research studies in the field of mobile-based health apps and the adoption of information technology have identified individual preferences and motivations to use these apps based on socioeconomic characteristics, demographics, access to health care facilities, perceptions about the usefulness of the apps, and the effect of existing or perceived disease conditions [15-18].

Hence, a cross-sectional analysis of the data generated from the app was performed to determine use trends of AYUSH measures by the public during the COVID-19 pandemic.

The primary objective of the cross-sectional analysis was to determine the extent of use of AYUSH advocacies and measures by the public for maintenance of health during the COVID-19 pandemic, as reported through the AYUSH Sanjivani mobile app.

Secondary objectives were to compare the self-reported incidence of COVID-19 and symptomatic status of the respondents affected with COVID-19 among the users of AYUSH measures as compared to nonusers and to determine the pattern of use of AYUSH measures by users across India. Perceived change in general well-being in terms of appetite, bowel habits, sleep, stamina, and mental well-being among users of AYUSH measures, the relationship between the duration of the use of AYUSH measures and the incidence of COVID-19, and the relationship between the symptomatic status of COVID-19–positive respondents among users and nonusers of AYUSH-based measures were also included as secondary objectives.

## Methods

### Study Design

This is a cross-sectional analysis of data generated through the AYUSH Sanjivani mobile app. The MoA launched the AYUSH Sanjivani app to generate data on the acceptance and use of AYUSH advocacies and measures by the population and its possible impact on the maintenance of health during the COVID-19 pandemic. The content of the app is a self-reporting questionnaire intended for the public to report their preferences, patterns, and trends of use of the measures circulated through the AYUSH advisory released by the MoA for maintenance of health during the pandemic. Self-perceived impact on improvement in general health and the benefits of using AYUSH measures during the COVID-19 pandemic by the respondents were also recorded in the app. We followed the Strengthening the Reporting of Observational Studies in Epidemiology (STROBE) guidelines when reporting the findings [19].

### Informed Consent and Ethical Consideration

The study was approved by the Central Ethics Committee of Central Council for Research in Ayurvedic Sciences, Ministry of AYUSH, India (1-12/2020-CARICD/Tech/CEC). Upon downloading the app, before voluntary consent was obtained, the user was informed that the information they entered would only be used for research purposes and that their anonymity and confidentiality would be maintained. The users were also informed that by choosing to provide information in this app, they were making a valuable contribution to public health research in the country. It was made explicitly clear that by participating in the survey, users were voluntarily giving their consent to use the data for research purposes.

### Study Setting

The app was released through the Google Play store in May 2020 and was available for download across India. The data generated from respondents across all States and Union Territories of India during the period from May 4 to July 31, 2020, were used for the analysis.

### Participants

All the residents of India who possessed a smartphone, tablet, or other such device and who were willing to download the app and voluntarily provide the requisite information to the questionnaire in either English or Hindi were eligible to participate in the study. The primary respondents were health seekers of AYUSH or their families, who preferred AYUSH systems for preventive or curative purposes; health seekers who sought consultation at the outpatient department of a national institution, research council, college, hospital, or primary or secondary health care facility across the country; and members of the public who were motivated to use AYUSH measures for maintenance of health during the COVID-19 pandemic. These beneficiaries were sensitized to the AYUSH measures through interaction with health professionals.

### Data Sources and Data Collection Methods

The AYUSH Sanjivani app was conceived to motivate and persuade the public to achieve a state of general well-being during the COVID-19 pandemic while documenting the patterns and trends of the use of AYUSH systems in India. The app was also announced and promoted through social media platforms such as the Twitter accounts, Instagram, and Facebook pages of MoA, AYUSH institutions, and hospitals, as well as AYUSH professionals and students, which enabled a wider reach among the general public. The AYUSH Sanjivani app was available for the Android and iOS (Apple) platforms and was made available through the App Store and Google Play store, and the questionnaire was drafted in a simple, easily comprehensible manner, initially launched in English and later rolled out in Hindi according to the World Health Organization guidance for translation and adaptation of instruments [20]. The app started with a COVID-19 guide for users that elaborated the importance of AYUSH for health, the need for self-care, and general and AYUSH measures to practice to improve immunity and maintain health. The Welcome screen of the app is shown in Multimedia Appendix 1.

The app contains three modules to report the desired data (Multimedia Appendix 2). The first module comprises a questionnaire for capturing the trends of use of AYUSH measures among the public across different geolocations. The second module is intended to capture the use trends of AYUSH measures by physicians. The third module aims to garner the cumulative data of health seekers and beneficiaries who were advised to use AYUSH preventive measures by their physicians. This paper focuses on the data collected through the first module of the app, which pertains to use trends of AYUSH preventive measures self-reported by the public. The questionnaire in the app was finalized by collaborative discussions with experts, followed by iterative refining, and included multiple choice questions, modified Likert scales, and yes/no questions. There were no open-ended questions. The questions were drafted in a simple, easy-to-understand format in English and Hindi so that they were comprehensible to people with basic education and knowledge of how to use smartphones.

The questionnaire was subdivided into three different layers. The first layer captured basic sociodemographic characteristics, such as gender, and the geographical location of the respondents. A question on whether they were using AYUSH measures (advised by the MoA or State governments, or other AYUSH measures) or not using any AYUSH measures was also included to classify the respondents into two categories (users or nonusers of AYUSH measures). Those who responded that they are using or have used AYUSH measures during this pandemic were asked three additional questions. The first question was related to the duration of use of AYUSH measures, and the second question captured the opinions of the respondents on whether the practice of AYUSH measures had benefitted them. The third question was intended to capture the possible reasons for finding AYUSH beneficial, reported as per the respondent’s experiences.

The second layer of the app contained another four questions, which could be answered by all respondents irrespective of whether they were using AYUSH measures. The questions were intended to capture the information such as the respondent’s occupation, presence or absence of any pre-existing disease, the risk of contracting COVID-19 (“at risk” was categorized as being in quarantine, a health care worker treating COVID-19 patients at hospitals or in communities, a general public official implementing lockdown, or a primary contact of a COVID-19–positive patient). The COVID-19 test status was elicited through a separate question in which the respondent was required to select any of the options of tested positive and asymptomatic, tested positive and symptomatic, tested negative, and never tested. The respondents were required to furnish data related to their COVID-19 status if they underwent testing either on their own or on medical advice.

The third layer of the app was accessible to only those respondents who were using AYUSH measures, and the questions included in it pertained to the use trends of measures advised under various AYUSH systems. This layer contained another set of six questions, namely duration of intake of AYUSH measures, the regularity of intake, self-perceived improvement in parameters of well-being (appetite, bowel habits, sleep, stamina, and mental well-being) or no improvement after use of AYUSH, and the onset of any influenza-like-illness symptoms. The respondent’s self-perceived impact on their general health was also captured (see the detailed questionnaire in Multimedia Appendix 3).

### Outcome Measures

The primary outcome was to measure the extent of use of AYUSH measures by the respondents who reported they used or did not use AYUSH measures during the COVID-19 pandemic. Further, the patterns and extent of use were assessed as distributions across sociodemographic characteristics such as geographical location, gender, urban or rural location, and occupation.

Secondary outcomes were to compare the incidence of COVID-19 among the respondents who did or did not use AYUSH measures, the pattern of use in terms of duration, regularity, use trends across different AYUSH systems, and the extent of benefits received assessed through a 5-point Likert scale ranging from strongly agree to strongly disagree. The reasons for finding AYUSH prophylactic measures beneficial in terms of responses were categorized as an overall feeling of good health, reducing the severity of symptoms while having COVID-19, or improvement in other minor ailments; these were also evaluated as a secondary outcome. Another secondary outcome was the overall improvement in the general health of the respondents based on the responses ranging from “no change” to “excellent improvement.” The change in parameters of well-being categorized as “improved or no change” elicited individually for all the parameters was also a secondary outcome. The association between the symptomatic status of respondents affected by COVID-19 and use or nonuse of AYUSH measures was evaluated. The association between the duration of use of AYUSH measures and incidence of COVID-19 was also evaluated as a secondary outcome.

### Bias

The app was promoted across social media platforms and through AYUSH institutions across the country for wider reach among all geographic and socioeconomic strata. However, the data are not representative of the population, as the respondents were restricted to people who are smartphone users and are more active on the web as well as those who already follow the MoA and other AYUSH-related pages on social media platforms. Moreover, the proportion of nonusers was much smaller compared to that of users; hence, the findings may have limited generalizability. The possibility of information bias could also not be completely ruled out, as the information provided in the app was retrospectively obtained, such as frequency of use, regularity of use, type of medicines used with the duration of use, etc.

### Study Size

Data from 723,459 respondents collected from May 4 to July 31, 2020, through the AYUSH Sanjivani mobile app was used for this cross-sectional analysis.

### Statistical Analysis

The qualitative data received through the app were imported into Excel (Microsoft Corporation), where they were numerically coded. Numerical codes were assigned to all the options for each question in the questionnaire. This coded Excel file was then imported into STATA 16.1 (StataCorp LLC) and used for statistical analysis. Descriptive statistics for categorical data were reported using frequencies and percentages. Comparisons among users and nonusers of AYUSH measures were performed using the chi-square test in terms of respondents being tested or not for COVID-19, the outcome of COVID-19 testing, the symptomatic status of the COVID-19–positive respondents, the risk status of the respondents, and the presence or absence of comorbid conditions. Logistic regression analysis was performed to compute the crude odds ratio by measuring the association between the duration of use of AYUSH-based measures and the outcome of COVID-19 testing (positive or negative). The association between the use of AYUSH-based measures and the symptomatic or asymptomatic status of COVID-19–positive respondents was also evaluated. Adjusted odds ratios considering the risk status and presence of comorbidities as confounders were also computed. A *P* value of <.05 was considered significant.

## Results

### Sociodemographic Profile of the Respondents

Data from 723,459 participants who downloaded the app and furnished the required information were available for analysis. The majority of the respondents (616,295/723,459, 85.2%) reported having been using AYUSH measures for maintenance of health during the pandemic. Among the 616,295 respondents who were AYUSH users, 493,766 (80.1%) were male, and 467,047 (75.8%) lived in rural areas. Data for occupation was available for 359,745 users, of which the majority were self-employed (123,274, 34.3%); 83,281 (23.2%) were students, and 67,230 (18.7%) were unemployed (Table 1).

**Table 1:** Demographic and basic characteristics of the respondents.

Characteristics		n (%)
**Used AYUSH^a^ measures during the COVID-19 pandemic (N=723,459)^b^**		
	Yes	616,295 (85.2)
	No	107,164 (14.8)
**Gender (n=616,295)^c^**		
	Male	493,766 (80.1)
	Female	122,188 (19.8)
	Did not wish to disclose	341 (0.1)
**Location (n=616,295)^c^**		
	Urban	148,888 (24.2)
	Rural	467,407 (75.8)
**Occupation (n=359,745)^d^**		
	Student	83,281 (23.2)
	Regular or salaried worker	33,989 (9.4)
	Casual worker	30,092 (8.4)
	Self-employed	123,274 (34.3)
	Unemployed	67,230 (18.7)
	Any other	21,879 (6.1)

^a^AYUSH: Ayurveda, yoga and naturopathy, Unani, Siddha, Sowa Rigpa, and homoeopathy.

^b^Distribution of total respondents.

^c^Distribution of respondents who were users of AYUSH-based measures.

^d^Distribution of users who responded to this question.

Among the 616,295 AYUSH users, the maximum participants were from Uttar Pradesh (158,053, 25.6%), followed by Maharashtra (86,328, 14.01%); Madhya Pradesh (57,894, 9.39%); Gujarat (46,815, 7.59%); and Chhattisgarh (44,461, 7.21%). A small proportion of the respondents were from the states of Rajasthan, Odisha, Haryana, Bihar, Karnataka, and West Bengal. The smallest number of respondents were from the states of Meghalaya and Manipur and the union territories of Ladakh and Lakshadweep (Multimedia Appendix 4).

### Use Trends Among Different Streams During the COVID-19 Pandemic

The number of respondents who reported using Ayurveda measures was 90,357/433,560 (21.0%), Homeopathy was used by 47,639/433,560 (11.0%), while a small proportion reported the use of Unani and Siddha Interventions. It is intriguing to note that 291,251/433,560 (67.0%) of the users reported having been using yoga, pranayama, meditation, or the use of home remedies such as spices in cooking, drinking warm water, steam inhalation, and other such practices for maintenance of health (Multimedia Appendix 5).

The use of warm water in routine life for drinking purposes was reported as the most commonly adopted measure, followed by the practice of yoga or pranayama as a choice for the maintenance of health and well-being. Among homeopathy medicines, Arsenicum Album 30C was the intervention of choice, while *Samshamani Vati* and AYUSH-64 were the most popular among the Ayurveda interventions. *Kaba Sura Kudineer*, a decoction used in the Siddha system, and the Unani interventions of *Behidana, Unnab*, and *Sapistan* decoction were reported as the most commonly used, albeit by only a small proportion of users. *Agastya Hareetaki* (an Ayurvedic intervention); use of *Anu Taila*, coconut oil, or sesame oil for nasal instillation, or oil pulling with coconut or sesame oil; use of *Chyavanprasha*; turmeric milk; and herbal tea were the other frequently used interventions in the Ayurveda stream. *Bryonia alba*, *Rhus toxicodendron*, *Belladonna*, *Gelsemium*, and *Eupatorium perfoliatum* were the other commonly used homeopathic interventions. *Nilavembu Kudineer* decoction and *Adathodai Manapagu* were some other popular Siddha interventions (Multimedia Appendix 6).

### Benefits Obtained by the Public Through the Use of AYUSH Measures

Among the 616,295 respondents who used AYUSH measures, 231,552 (37.5%) reported using them for more than 30 days, while 231,735 (37.6%) reported having used them for less than 15 days. A considerable proportion of respondents either strongly agreed (420,395/616,295, 68.2%) or moderately agreed (133,406/616,295, 21.6%) that the use of AYUSH measures had benefitted them. AYUSH measures were considered beneficial by 487466/616,295 (79.1%) of the respondents, as these measures gave them an overall feeling of good health. Most of the respondents (227,952/616,295, 63.6%) reported improvement in parameters of well-being in terms of their perception of change in appetite, bowel movements, sleep, stamina, and mental well-being. Improvement in appetite was reported by 20,618/359,785 respondents (5.7%), while improvement in bowel habits was reported by 42,017/359,785 respondents (11.7%). Moreover, improvement in more than one parameter was reported by 98,352/359,785 respondents (27.3%). Overall improvement in general health was rated as excellent by 30,848 of 359,785 respondents (8.6%), while 144,927 (40.3%) reported good improvement and 133,046 (36.9%) reported slight improvement (Table 2).

**Table 2:** Trends of use and benefits obtained by respondents who were users of AYUSH measures (n=616,295).

Characteristic		Value, n (%)
**Duration of use of AYUSH^a^ measures**		
	Less than 15 days	231,735 (37.6)
	15-30 days	153,008 (24.8)
	More than 30 days	231,552 (37.6)
**Opinion on whether the AYUSH prophylactic measures have benefitted them during the COVID-19 pandemic (n=616,295)**		
	Strongly agree	420,395 (68.2)
	Moderately agree	133,406 (21.6)
	Moderately disagree	11,309 (1.8)
	Strongly disagree	17,496 (2.8)
	Neutral/can’t say	33,689 (5.5)
**Reason for finding AYUSH prophylactic measures beneficial (n=616,295)**		
	Overall feeling of good health	487,466 (79.1)
	Helped prevent COVID-19 (respondent’s perception)	50,721 (8.2)
	Reduced symptoms while having COVID-19	31,057 (5.0)
	Helped improve minor ailment other than COVID-19	47,051 (7.6)
**Improved parameters of well-being (n=359,785)**		
	Appetite	20,618 (5.7)
	Bowel movements	42,017 (11.7)
	Sleep	13,559 (3.8)
	Stamina	26,397 (7.3)
	Mental well-being	27,009 (7.5)
	Improvement in more than one of the above parameters	98,352 (27.3)
	No change	131,833 (36.6)
**Overall improvement in general health (n=359,785)**		
	Excellent improvement	30,848 (8.6)
	Good improvement	144,927 (40.23)
	Slight improvement	133,046 (36.9)
	No change	18,240 (5.1)
	Unknown	32,724 (9.01)

^a^AYUSH: Ayurveda, yoga and naturopathy, Unani, Siddha, Sowa Rigpa, and homoeopathy.

### Data on Pre-existing Diseases, Symptoms, and Risk Status Among the Respondents

Data on pre-existing diseases (comorbidities) were furnished by 408,089 respondents, of whom 380,731 (93.3%) reported the absence of any pre-existing disease. Hypertension was the most common pre-existing disease (comorbidity), reported by 11,941/408,089 respondents (2.9%), followed by diabetes mellitus, heart disease, and asthma. The presence of more than one pre-existing disease was reported by 9266/408,089 respondents (2.3%) (Table 3).

**Table 3:** Comparison of COVID-19 test status, incidence, and symptomatic status among users and nonusers of AYUSH measures. Percentages are calculated based on the total for each column.

Characteristics		Total, n (%)	Respondents who were users of AYUSH^a^ measures, n (%)	Respondents who were nonusers of AYUSH measures, n (%)	*P* value^b^
**COVID-19 test status (n=408,089)**					.67
	Tested	27,661 (6.8)	24,368 (6.8)	3293 (6.8)	
	Not tested	380,428 (93.2)	335,469 (93.2)	44,959 (93.2)	
	Total	408,089 (100)	359,837 (100)	48,252 (100)	
**COVID-19 test result (n=27,661)**					<.001
	Positive	13,320 (48.2)	12,002 (49.3)	1318 (40.0)	
	Negative	14,341 (51.8)	12,366 (50.7)	1975 (60.0)	
	Total	27,661 (100)	24,368 (100)	3293 (100)	
**Symptomatic status of COVID-19 positive respondents (n=13,320)**					<.001
	Asymptomatic	8545 (64.2)	8100 (67.6)	445 (33.8)	
	Symptomatic	4775 (35.8)	3902 (32.5)	873 (66.2)	
	Total	13,320 (100)	12,002 (100)	1318 (100)	
**Risk category (n=408,089)**					<.001
	In risk category	19,757 (4.8)	16,583 (4.6)	3174 (6.6)	
	Not in risk category	388,332 (95.2)	343,254 (95.4)	45,078 (93.4)	
	Total	408,089 (100)	359,837 (100)	48,252 (100)	
**Comorbid conditions (n=408,089)**					<.001
	Hypertension	11,941 (2.9)	10,638 (3.0)	1303 (2.7)	
	Asthma	1089 (0.3)	413 (0.1)	676 (1.4)	
	Diabetes mellitus	2772 (0.7)	2284 (0.6)	488 (1.0)	
	Heart disease	1230 (0.3)	992 (0.3)	238 (0.5)	
	Kidney disease	671 (0.2)	490 (0.1)	181 (0.4)	
	Cancer	389 (0.1)	234 (0.1)	155 (0.3)	
	Presence of multiple comorbid conditions	9266 (2.3)	8033 (2.2)	1233 (2.5)	
	None	380,731 (93.3)	336,753 (93.6)	43,978 (91.2)	

^a^AYUSH: Ayurveda, yoga and naturopathy, Unani, Siddha, Sowa Rigpa, and homoeopathy.

^b^Compared using chi-square tests.

### COVID-19 Status Among Respondents

Among 408,089 participants, only 27,661 (6.8%) underwent testing for COVID-19. Out of the 27,661 respondents who underwent testing, 13,320 (48.2%) tested positive for COVID-19, among whom 8545 (64.2%) reported themselves to be asymptomatic.

### Duration of Use of AYUSH and Symptom Status of COVID-19–Positive Respondents

Among the 12,002 respondents who tested positive for COVID-19 and used AYUSH measures, 8101 (67.5%) reported their duration of use of AYUSH measures as less than 30 days, and the others reported a longer duration of use. Among the 12,002 COVID-19–positive respondents using AYUSH measures, 8100 (67.5%) were asymptomatic.

### Association Between Duration of Use of AYUSH Measures and Incidence of COVID-19

The results of the logistic regression analysis depict that the odds ratio (OR) of testing positive for COVID-19 is 1.52 (95% CI 1.44-1.60, *P*<.001) for respondents who were using AYUSH measures for less than 30 days compared to those who were using these measures for more than 30 days. The adjusted OR considering the effects of confounders, namely the presence of comorbidities and the respondent being in a risk category, is 0.90 (95% CI 0.85-0.95, *P*<.001) (Table 4).

**Table 4:** Logistic regression analysis to identify the association between the duration of use of AYUSH-based measures and the outcome of COVID-19 testing.

Variable		COVID-19–positive (n=12,002)	COVID-19–negative (n=12,366)
**Duration of use of AYUSH^a^-based measures^b,c^**			
	More than 30 days	3901 (32.5)	5221 (42.2)
	Less than 30 days	8101 (67.5)	7145 (57.8)
**Comorbidities**			
	Absent	5679 (47.3)	10131 (81.9)
	Present	6323 (52.7)	2235 (18.1)
**Risk category**			
	Not in a risk category	4866 (40.5)	10203 (82.5)
	In a risk category	7136 (59.5)	2163 (17.5)

^a^AYUSH: Ayurveda, yoga and naturopathy, Unani, Siddha, Sowa Rigpa, and homoeopathy.

^b^Crude odds ratio (95% CI) 1.52 (1.44-1.60). The crude odds ratio was computed through univariate logistic regression analysis by taking the outcome of the COVID-19 test (positive or negative) as the dependent variable and the duration of use of AYUSH measures as the independent variable.

^c^Adjusted odds ratio (95% CI) 0.90 (0.85-0.95). The adjusted odds ratio was computed through multivariate analysis adjusted for the presence of comorbidities and risk category as confounders.

### Association Between the Use of AYUSH Measures and Symptomatic Status of COVID-19 Respondents

The results of the logistic regression analysis revealed that the OR of being symptomatic was 4.01 (95% CI 3.61-4.59) for nonusers of AYUSH measures compared to users. The adjusted OR considering the effects of confounders, namely the presence of comorbidities and respondents being in a risk category, is 3.48 (95% CI 3.06-3.95) (Table 5).

**Table 5:** Logistic regression analysis to identify the association between use of AYUSH-based measures and symptomatic status of respondents who tested positive for COVID-19.

Variable		Symptomatic COVID-19–positive participants (n=4775)	Asymptomatic COVID-19–positive participants (n=8545)
**Used AYUSH^a^-based measures^b,c^**			
	Yes	3902 (81.7)	8100 (94.8)
	No	873 (18.3)	445 (5.2)
**Comorbidities**			
	Absent	1576 (33.0)	4499 (52.6)
	Present	3199 (67.0)	4046 (47.4)
**Risk category**			
	Not in a risk category	785 (16.7)	4345 (50.8)
	In a risk category	3900 (83.3)	4200 (49.2)

^a^AYUSH: Ayurveda, yoga and naturopathy, Unani, Siddha, Sowa Rigpa, and homoeopathy.

^b^ Crude odds ratio (95% CI) 4.01 (3.61-4.59). The crude odds ratio was computed through univariate logistic regression analysis by taking the symptomatic status of COVID-19–positive respondents as the dependent variable and use of AYUSH-based measures as an independent variable without adjusting for confounders.

^c^Adjusted odds ratio (95% CI) 3.48 (3.06-3.95). The adjusted odds ratio was computed through multivariate analysis adjusted for the presence of comorbidities and risk category as confounders.

## Discussion

### Principal Findings

A representative population of 723,459 people from different geolocations across the country downloaded the AYUSH Sanjivani app and reported the perceptions and practices they had adopted in the wake of the COVID-19 pandemic that significantly altered their lifestyle. A majority of the respondents used AYUSH measures for maintenance of health and prevention of disease, and most of them reported having benefitted from the use of various interventions and practices. A positive association between the prolonged practice of AYUSH measures and symptomatic status could be observed in the respondents who were infected with COVID-19.

In this study, the maximum representation was of AYUSH users, and it is expected that the willingness to use an app specifically targeting AYUSH users will be greater among health seekers who are familiar with these systems of medicine. Findings from a previous study revealed that even in a developing country such as India, 32% of the patients attending a medical care facility in urban settings used the internet, and 75% of them sought medical information through the internet; this would support the substantial amount of data generated through this app [21]. In a national representative cross-sectional survey conducted in 2014 in India, it was observed that 6.9% of all patients sought AYUSH services for different ailments in a recall period of 2 weeks, without a great differential between urban and rural regions [22]. This targets the reported use of AYUSH care services for disease management, which is expected to be lower compared to the use of the AYUSH system for preventive care.

Maximum reporting was observed from the states of Uttar Pradesh, Maharashtra, and Madhya Pradesh, which can be attributed to the high population density in these states. The decision to seek health care is not only contingent upon the experience of illness but also depends on various social, economic, and demographic factors [22]; it was observed that approximately three quarters of the total respondents were from rural areas, and most of them were users of AYUSH measures. This can be attributed to the tendency of people in rural areas to adhere more to tradition compared to the urban population. This is consistent with a study based on the World Health Organization Study on Global Ageing and Adult Health (WHO-SAGE) survey, which suggests that individuals living in rural areas are more likely to report the use of traditional healers [23]. In this study, it was observed that in the setting of India, being female was associated with a lower likelihood of users downloading mobile apps and furnishing their personal information and preferences, despite evidence from recent studies that does not suggest differential internet use between males and females [17]. Students and self-employed workers accounted for the majority of respondents as well as of users of AYUSH measures, which underlines the findings of earlier studies that predict that the younger population, literate people, and full-time workers are more likely to use health apps and be motivated to use health-related advice [24].

The majority of respondents reported having benefitted from using AYUSH measures; they rated the degree of improvement as mild, good, or excellent, and attributed this improvement to their perceived experience of overall well-being. The self-reported public experience of improvement in parameters of well-being, such as sleep, appetite, stamina, mental well-being, and sleep, is a good indicator for integrating AYUSH measures for well-being into the daily routine. Preliminary evidence on the impact of COVID-19 on the public reveals significant health-related anxiety, generalized anxiety, psychological stress, and sleep disorders, and government-implemented lockdowns inculcated many habits, such as decreased physical activity and exercise and increased snacking, with deleterious effects on vulnerable populations and especially on those with pre-existing comorbidities [25,26].

The improvements perceived in the level of well-being and the general aspects of health measured in terms of individual satisfaction with appetite, sleep, stamina, mental wellness, and bowel habits indicate a positive role for the use of traditional AYUSH interventions and practices in maintaining holistic health and preventing long-lasting adverse health outcomes.

Ayurveda and homeopathy were the systems of medicine that were preferred by the majority of the respondents; this can be attributed to the maximum number of hospitals and health care providers in India under these two AYUSH systems. Arsenicum Album 30C, *Samshamani Vati*, and Ayush-64, which were deployed as frontline prophylactic interventions, were the most used interventions by the public to maintain health. Arsenicum Album 30C is a homeopathic intervention that is used for respiratory ailments; meanwhile, the other two interventions are Ayurvedic formulations (*Samshamani Vati* and Ayush-64), which are prescribed for the clinical management of pyrexia, influenza-like illness, cough, and dyspnea [27].

The practices that the public engaged in include the practice of yoga, pranayama, and meditation along with common home remedies such as using spices in cooking, drinking turmeric milk, drinking warm water, and steam inhalation. The practice of engaging the mind and body through meditation, pranayama, and yoga has attracted significant attention and has been extensively studied for its possible beneficial effects on physical and mental health outcomes [28]. A growing body of evidence suggests that the elements of physical postures, breathing, and meditation can improve physical well-being, including balance, range of motion, blood pressure, pain, fatigue, and general health, which could be correlated with the benefits reported by the AYUSH users [29].

The proportion of participants who underwent laboratory testing for COVID-19 among AYUSH users and nonusers could not be compared to arrive at meaningful outcomes, as the majority of the respondents were using AYUSH measures, and those not using it were few in number. A longer duration of use of AYUSH measures is more likely to produce better protection when compared with use for less than 15 days, as it was observed that the likelihood of being COVID-19–positive was lower in respondents who were using the AYUSH measures for more than 30 days. In AYUSH systems, the use of diet or medicine is targeted at producing an ideal state of homeostasis, which would reflect the inherent strength of the body in immunopotentiation and prevention of diseases. Clinical studies demonstrating *Rasayana* activity (medicines or practices with rejuvenating potential) in healthy individuals reflect better outcomes when administered for 60 days or more, which implies that compliance with AYUSH measures would ideally require a longer duration to act in the macrochannels and microchannels of circulation to bring about optimal health; this underlines the pattern seen in this analysis, where the odds of being COVID-19–positive or symptomatic for respondents who tested positive are greater in respondents who have used the preventive measures for a lesser duration [30]. Moreover, a good proportion of the respondents who used AYUSH measures used home remedies, yoga, pranayama, meditation, and other practices without resorting to the use of any AYUSH medications with specific prophylactic potential.

The qualitative appraisal of the analyzed data reflects that a considerable majority of the respondents benefitted from the use of AYUSH measures, which were either traditional formulations with centuries of use in the maintenance of health or home remedies and are an integral part of Indian culture and cuisine. Due to the long history of use of many herbal remedies and the experiences that have passed from generation to generation, people are relying on herbal remedies and some simple home remedies for common diseases that are used across India irrespective of sociocultural, religious, and geographical differences [31]. The use of AYUSH measures is likely to evoke a positive response to the psychological and physical well-being of the respondents [32].

### Limitations and Strengths of the Study

Because this is a cross-sectional analysis of the data generated from a mobile app, the documented data are a representation of smartphone users only. A limitation of the study is the inability to capture generalizable data reflecting true health-seeking trends, as only people with access to smartphones and good internet connectivity responded to the questionnaire. Because the representation of nonusers of AYUSH measures was minimal, statistical comparison among users and nonusers of AYUSH measures could not be performed. Although AYUSH measures are generally practiced in many states for both curative as well as preventive aspects, representation from some of these states was meager; hence, a true representation of users or nonusers of AYUSH measures could not be captured. The incidence of COVID-19 among the respondents was self-reported, and it is difficult to determine the relationship between the use of AYUSH measures, duration of use, and incidence of disease or symptomatic status among the general public.

Finally, this is the first study to document how time-tested indigenous systems of medicine are being used by the public during a pandemic of unprecedented spread, morbidity, and mortality. The large amount of data obtained is the greatest benefit of this analysis, as it would sweep outliers that may misrepresent the data and has enabled us to provide a realistic picture of the characteristic attributes and patterns of the population. This analysis offers a starting point for future researchers to initiate more interventional studies based on the use trends demonstrated in this study.

### Conclusion

The findings of the cross-sectional analysis assert that a good proportion of the representative population has practiced AYUSH advocacy across different geolocations of the country during the COVID-19 pandemic. Although anecdotally, people report that traditional systems of holistic healing are good for the maintenance of health and well-being, our study findings also support that use of AYUSH measures provided better health, improved parameters of well-being, and even helped prevent other illnesses. This pattern suggests possibilities of exploring the role of AYUSH care, considering its acceptance, accessibility, and possible benefits, in the area of pluralistic health care.

To improve the use of a pluralistic health care delivery system, it is imperative to understand the acceptability, use trends, and possible impact on the quality of life and specific domains of health among the public. The response obtained in this study points to a possible functional integration and cross-hybridization of the merits of different systems to effectively generate a positive outcome on integrated health care delivery targeting universal health coverage. To assess the multiple levels of impact of AYUSH preventive measures on health, future studies need to apply diverse disciplines and methods, including intervention studies, longitudinal cohort studies, as well as qualitative observations to examine the nature of the benefits offered by these measures.

## Appendix

Multimedia Appendix 1 Welcome screen of the app.

Multimedia Appendix 2 Modules and layers of the app.

Multimedia Appendix 3 Detailed questionnaire.

Multimedia Appendix 4 Statewise distribution of the respondents.

Multimedia Appendix 5 Distribution of respondents as per their choice of AYUSH streams for prevention.

Multimedia Appendix 6 Distribution of the respondents as per the choice of AYUSH interventions and the duration of use.

## Abbreviations

AYUSH: Ayurveda, yoga and naturopathy, Unani, Siddha, and homeopathy
HCoV: human coronavirus
IT: information technology
MoA: Ministry of AYUSH
SARS-CoV: severe acute respiratory syndrome coronavirus
STROBE: Strengthening the Reporting of Observational Studies in Epidemiology
WHO-SAGE: World Health Organization Study on Global Ageing and Adult Health
