# Supplementary figures and images for: Mobile App–Reported Use of Traditional Medicine for Maintenance of Health in India During the COVID-19 Pandemic: Cross-sectional Questionnaire Study
Source: JMIRx Med. 2021 May 7;2(2):e25703. doi: 10.2196/25703 (PMC8110045; doi:10.2196/25703)

## Multimedia Appendix 1: Welcome screen of the AYUSH Sanjivani app

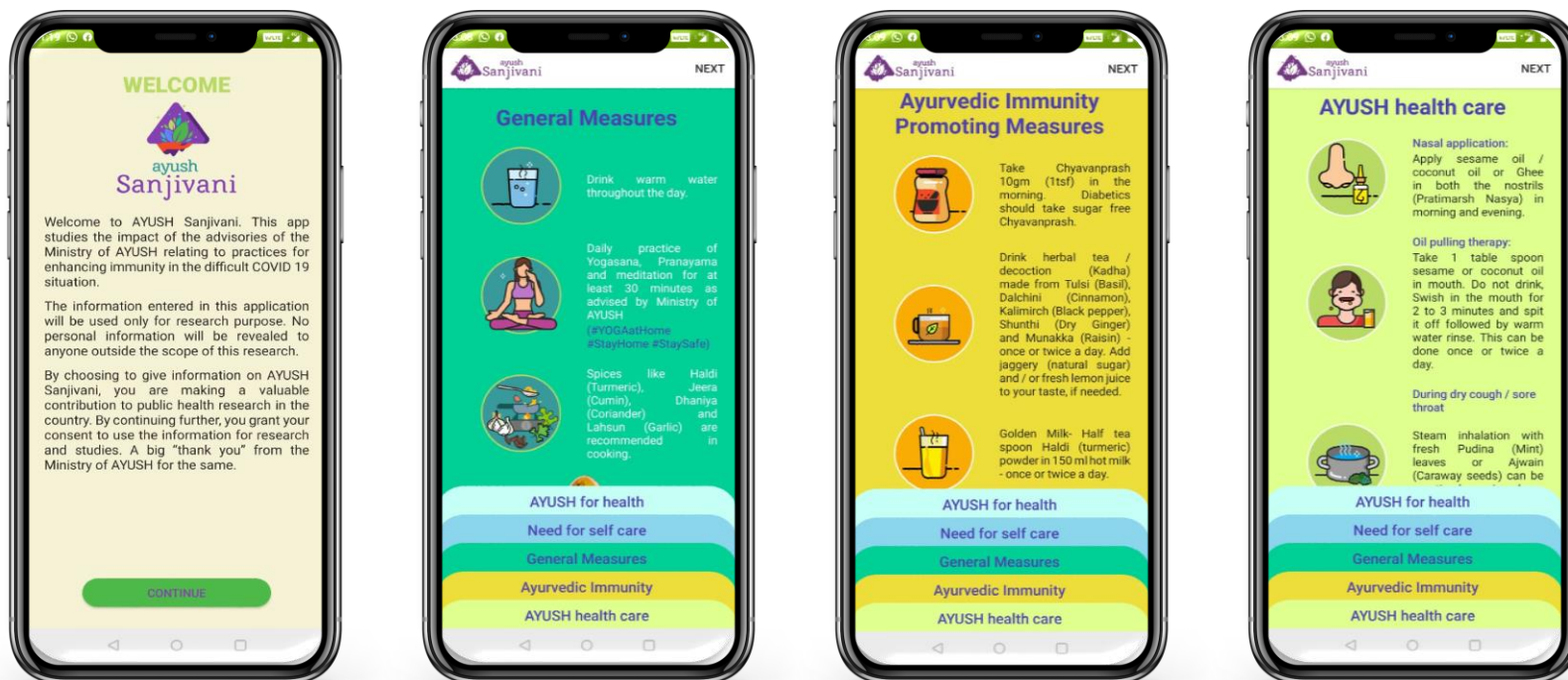

Supplement: Multimedia Appendix 1 [file xmed_v2i2e25703_app1.pdf]

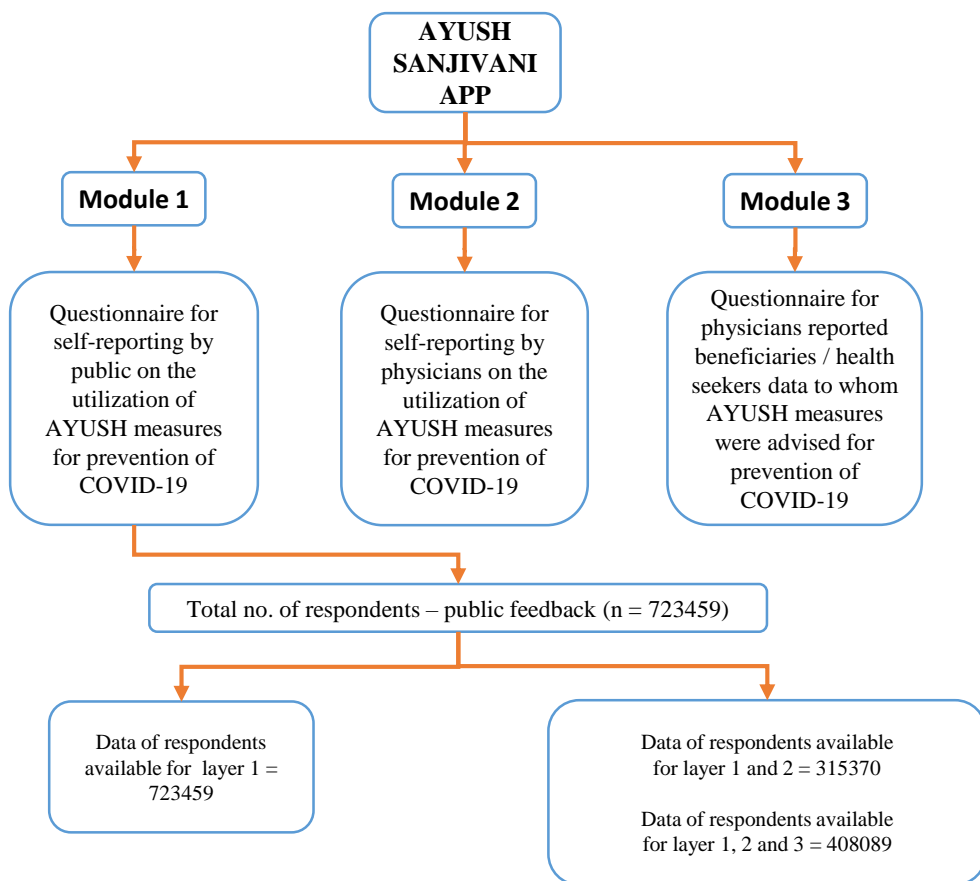

**Multimedia Appendix 2: Modules and layers of the app**

Supplement: Multimedia Appendix 2 [file xmed_v2i2e25703_app2.pdf]

## Multimedia Appendix 3: Detailed questionnaire

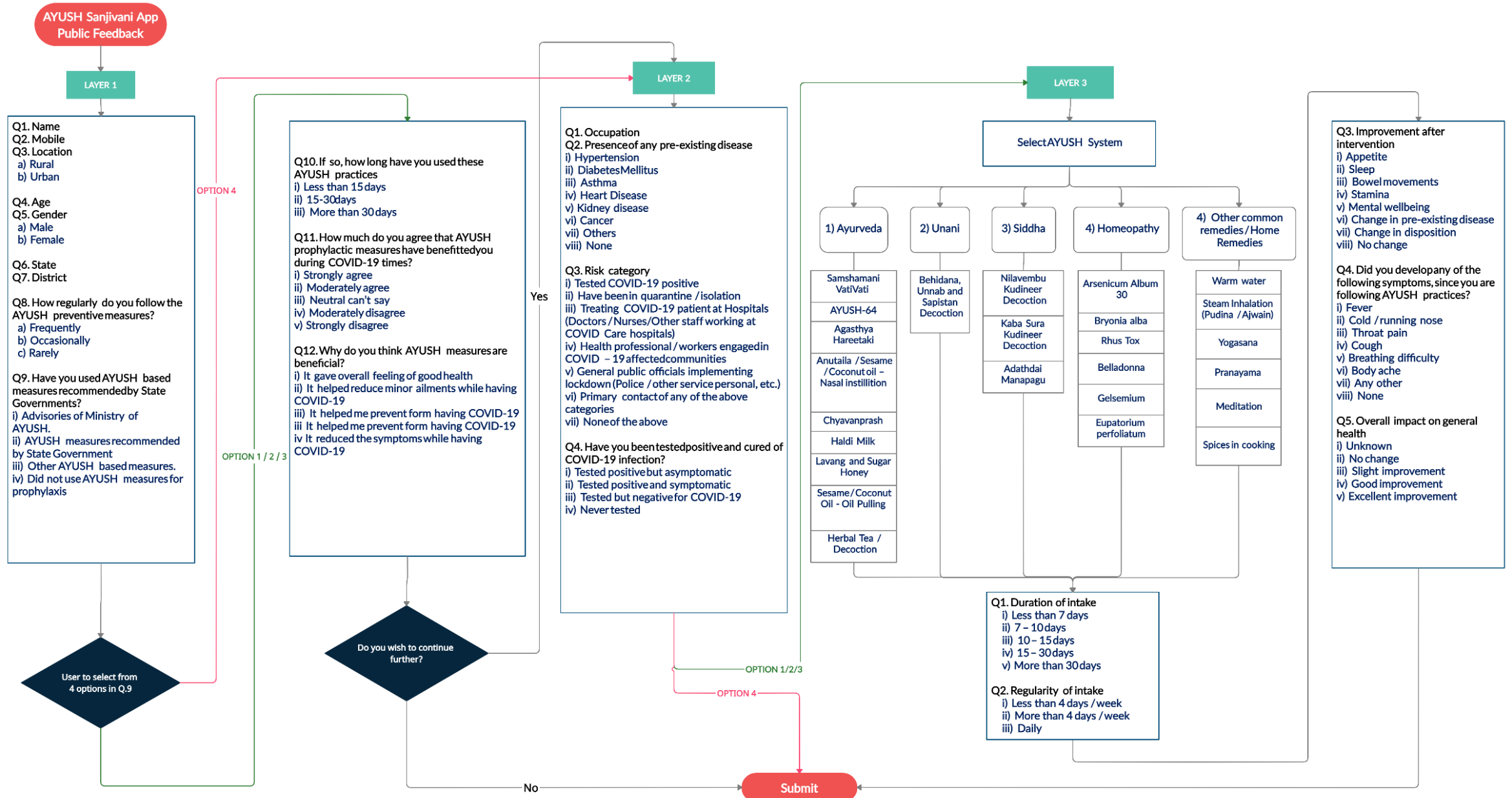

Supplement: Multimedia Appendix 3 [file xmed_v2i2e25703_app3.pdf]
