# Supplementary material for: Mobile App–Reported Use of Traditional Medicine for Maintenance of Health in India During the COVID-19 Pandemic: Cross-sectional Questionnaire Study
Source: JMIRx Med. 2021 May 7;2(2):e25703. doi: 10.2196/25703 (PMC8110045; doi:10.2196/25703)
Supplement: Multimedia Appendix 4 [file xmed_v2i2e25703_app4.pdf]

Multimedia Appendix 4: State wise distribution of the respondents

State / UTs	Total	Respondents who use AYUSH measures for prevention of COVID-19	Respondents who did not used AYUSH measures for prevention of COVID-19
**Uttar Pradesh**	178550	158053 (88.5%)	20497 (11.5%)
**Maharashtra**	98569	86328 (87.6%)	12241 (12.4%)
**Madhya Pradesh**	64386	57894 (89.9%)	6492 (10.1%)
**Gujarat**	51857	46815 (90.3%)	5042 (9.7%)
**Chhattisgarh**	50057	44461 (88.2%)	5596 (11.2%)
**Rajasthan**	33413	28332 (84.8%)	5081 (15.2%)
**Odisha**	33083	26302 (79.5%)	6781 (20.5%)
**Haryana**	30709	24656 (80.3%)	6053 (19.7%)
**Bihar**	29398	24267 (82.5%)	5131 (17.5%)
**Karnataka**	28582	18439 (64.5%)	10143 (35.5%)
**West Bengal**	23710	20970 (88.4%)	2740 (11.6%)
**Assam**	13391	11406 (85.2%)	1985 (14.8%)
**Punjab**	12792	11276 (88.1%)	1516 (11.9%)
**Jharkhand**	10522	8521 (81.0%)	2001 (19.0%)
**Tamil Nadu**	10371	7739 (74.6%)	2632 (25.4%)
**Uttarakhand**	10149	8869 (87.4%)	1280 (12.6%)
**Delhi**	8627	5323 (61.7%)	3304 (38.3%0
**Jammu & Kashmir**	7123	5456 (76.6%)	1667 (23.4%)
**Himachal Pradesh**	6442	5234 (81.2%)	1208 (18.8%)
**Kerala**	6036	4648 (77.0%)	1388 (23.0%)
**Andhra Pradesh**	4542	3172 (69.8%)	1370 (30.2%)
**Telangana**	4199	2687 (64.0%)	1512 (36.0%)
**Tripura**	3718	3256 (87.6%)	462 (12.4%)
**Mizoram**	1324	1001 (75.6%)	323 (24.4%)
**Nagaland**	367	164 (44.7%)	203 (55.3%)
**Goa**	346	244 (70.5%)	102 (29.5%)
**Sikkim**	278	196 (70.5%)	82 (29.5%)
**Chandigarh**	197	116 (58.9%)	81 (41.1%)
**Arunachal Pradesh**	174	103 (59.2%)	71 (40.8%)
**Andaman & Nicobar**	129	81 (62.8%)	48 (37.2%)
**Puducherry**	126	94 (74.6%)	32 (25.4%)
**Daman & Diu**	99	72 (72.7%)	27 (27.3%)
**Dadra & Nagar Haveli**	76	53 (69.7%)	23 (30.3%)
**Meghalaya**	57	34 (59.6%)	23 (40.4%)
**Manipur**	51	30 (58.8%)	21 (41.2%)
**Ladakh**	7	2 (28.6%)	5 (71.4%)
**Lakshadweep**	2	1 (50.0%)	1 (50.0%)
**Grand Total**	**723459**	**616295 (85.2%)**	**107164 (14.8%)**

Values have been given as n (%)
