# Supplementary material for: Mobile App–Reported Use of Traditional Medicine for Maintenance of Health in India During the COVID-19 Pandemic: Cross-sectional Questionnaire Study
Source: JMIRx Med. 2021 May 7;2(2):e25703. doi: 10.2196/25703 (PMC8110045; doi:10.2196/25703)

Multimedia Appendix 5: Distribution of respondents as per their choice of AYUSH system for maintenance of health

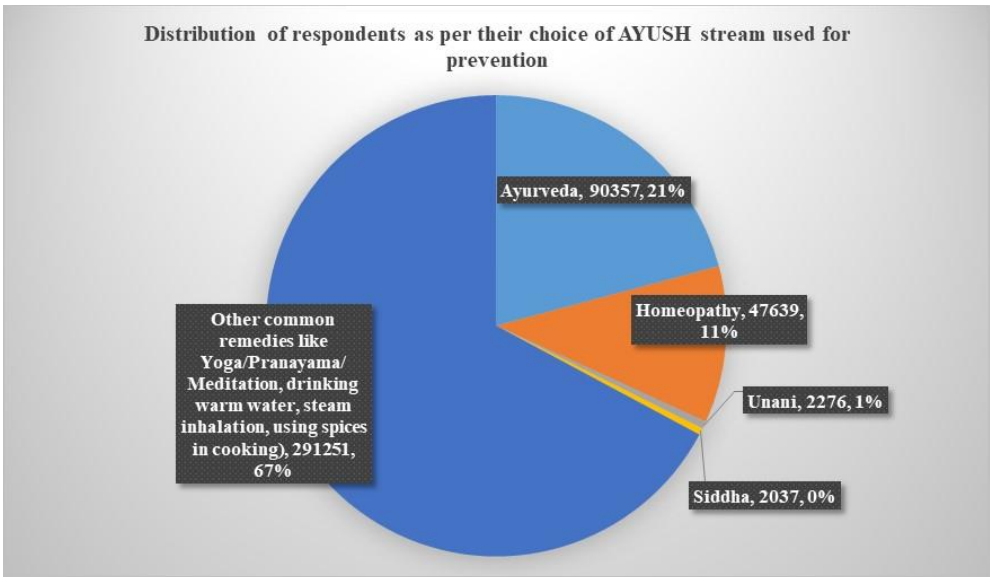

Supplement: Multimedia Appendix 5 [file xmed_v2i2e25703_app5.pdf]
