# Supplementary material for: Mobile App–Reported Use of Traditional Medicine for Maintenance of Health in India During the COVID-19 Pandemic: Cross-sectional Questionnaire Study
Source: JMIRx Med. 2021 May 7;2(2):e25703. doi: 10.2196/25703 (PMC8110045; doi:10.2196/25703)
Supplement: Multimedia Appendix 6 [file xmed_v2i2e25703_app6.pdf]

Multimedia Appendix 6: Distribution of the respondents as per their choice of AYUSH interventions / measures and the duration of use

Intervention	Duration of use				
	Less than 7 days	7-10 days	10-15 days	15-30 days	More than 30 days
**Samshamani Vati (n = 42970)**	5968 (13.9)	1570 (3.7)	1679 (3.9)	6074 (14.1)	27679 (64.4)
**AYUSH-64 (n=44882)**	6343 (14.1)	2206 (4.9)	2336 (5.2)	4847 (10.8)	29150 (64.9)
**Agastya Hareetaki (n=27923)**	2071 (7.4)	591 (2.1)	805 (2.9)	2290 (8.2)	22166 (79.4)
**Anuthaila / Seasame / coconut oil (n=33398)**	22756 (68.1)	934 (2.8)	1355 (4.1)	3927 (11.8)	4426 (13.3)
**Chyavanprash (n=6494)**	913 (14.1)	335 (5.2)	497 (7.7)	1511 (23.3)	3238 (49.9)
**Haldi Milk (n=18777)**	2664 (14.2)	1153 (6.1)	1514 (8.1)	4037 (21.5)	9409 (50.1)
**Lavang and Sugar Honey (n=13150)**	1856 (14.1)	692 (5.3)	1034 (7.9)	4073 (31.0)	5495 (41.8)
**Seasame / Coconut Oil - Nasal (n=5663)**	1171 (20.7)	343 (6.1)	518 (9.1)	1690 (29.8)	1941 (34.3)
**Seasame / Coconut Oil - Oil Pulling (n=5363)**	1099 (20.5)	313 (5.8)	477 (8.9)	1682 (31.4)	1792 (33.4)
**Herbal Tea / Decoction (11454)**	1583 (13.8)	690 (6.0)	993 (8.7)	2671 (23.3)	5517 (48.2)
**Arsenicum Album 30 (n=45090)**	4271 (9.5)	853 (1.9)	1821 (4.0)	29378 (65.2)	8767 (19.4)
**Bryonia alba (n=3709)**	683 (18.4)	245 (6.6)	293 (7.9)	1394 (37.6)	1094 (29.5)
**Rhus Tox (n=3836)**	731 (19.1)	254 (6.6)	337 (8.8)	1368 (35.7)	1146 (29.9)
**Belladonna (n=3494)**	655 (18.7)	203 (5.8)	302 (8.6)	1368 (39.2)	966 (27.6)
**Gelsemium (n=3238)**	546 (16.9)	177 (5.5)	294 (9.1)	1342 (41.4)	879 (27.1)
**Eupatorium perfoliatum (n=3101)**	515 (16.6)	158 (5.1)	297 (9.6)	1299 (41.9)	832 (26.8)

**Behidana, Unnab and Sapistan Decoction (n=2274)**	426 (18.7)	333 (14.6)	707 (31.1)	390 (17.2)	418 (18.4)
**Nilavembu Kudineer Decoction (n=1302)**	425 (32.6)	141 (10.8)	153 (11.8)	151 (11.6)	432 (33.2)
**Kaba Sura Kudineer Decoction (n=1488)**	418 (28.1)	220 (14.8)	193 (13.0)	230 (15.5)	427 (28.7)
**Adathdai Manapagu (n=863)**	242 (28.0)	82 (9.5)	112 (13.0)	135 (15.6)	292 (33.8)
**Warm water (n = 248888)**	22324 (9.0)	9676 (3.9)	14412 (5.8)	67953 (27.3)	134523 (54.0)
**Steam Inhalation (Pudina / Ajwain) (n = 11346)**	2216 (19.5)	953 (8.4)	1255 (11.1)	3348 (29.5)	3574 (31.5)
**Yogasana (n = 79207)**	9312 (11.8)	3459 (4.4)	4545 (5.8)	11382 (14.4)	50509 (63.9)
**Pranayama (n = 44900)**	5413 (12.1)	864 (1.9)	1307 (2.9)	3637 (8.1)	33679 (75.0)
**Meditation (n=12205)**	2014 (16.5)	738 (6.0)	1190 (9.8)	2911 (23.9)	5352 (43.9)
**Spices in cooking (n=17556)**	2200 (12.5)	898 (5.1)	1320 (7.5)	3833 (21.8)	9305 (53.0)
**Values have been reported as n (%)**					
